# Optimal parameters for laccase-mediated destaining of Coomassie Brilliant Blue R-250-stained polyacrylamide gels

**DOI:** 10.1016/j.dib.2016.01.029

**Published:** 2016-01-29

**Authors:** Jie Yang, Xiaodan Yang, Xiuyun Ye, Juan Lin

**Affiliations:** Fujian Key Laboratory of Marine Enzyme Engineering, Fuzhou University, Fuzhou, Fujian 350116, China

**Keywords:** ABTS, 2,2′-azino-bis (3-ethylbenzothiazoline-6-sulfonate), ACE, acetosyringone, BSA, bovine serum albumin, CBBR, Coomassie Brilliant Blue R-250, HBT, 1-hydroxybenzotriazole, SYA, syringic acid, SYD, syringaldehyde, Laccase, Destaining, Polyacrylamide gel, Coomassie Brilliant Blue R-250

## Abstract

The data presented in this article are related to the research article entitled “Destaining of Coomassie Brilliant Blue R-250-stained polyacrylamide gels with fungal laccase” [1]. Laccase is a class of multicopper oxidases that can catalyze oxidation of recalcitrant dyestuffs. This article describes optimal parameters for destaining of polyacrylamide gels, stained with Coomassie Brilliant Blue R-250, with laccase from basidiomycete *Cerrena* sp. strain HYB07. Effects of laccase activity, mediator type and concentration, temperature and time on destaining of polyacrylamide gels were evaluated with respect to gel background intensity and protein band signals, and the optimal destaining effects were obtained with 15 U mL^−1^ laccase and 2 μM ABTS at 37 °C after 2 h.

## Specifications table

**Table:** 

Subject area	Biology
More specific subject area	Electrophoresis
Type of data	Figures
How data was acquired	Photography
Data format	Analyzed
Experimental factors	Crude laccase of *Cerrena* sp. HYB07 was used
Experimental features	CBBR-stained polyacrylamide gels were destained with laccase/ABTS
Data source location	Fuzhou University, Fuzhou, China
Data accessibility	Data accessibility

## Value of the data

● Application of laccase in polyacrylamide gel destaining was described.
● This is the first report on parameter optimization of laccase-mediated destaining of polyacrylamide gels.
● The data provided application guidelines for other laccases to be used in polyacrylamide gel destaining.

## 1 Data

**Fig. 1:**
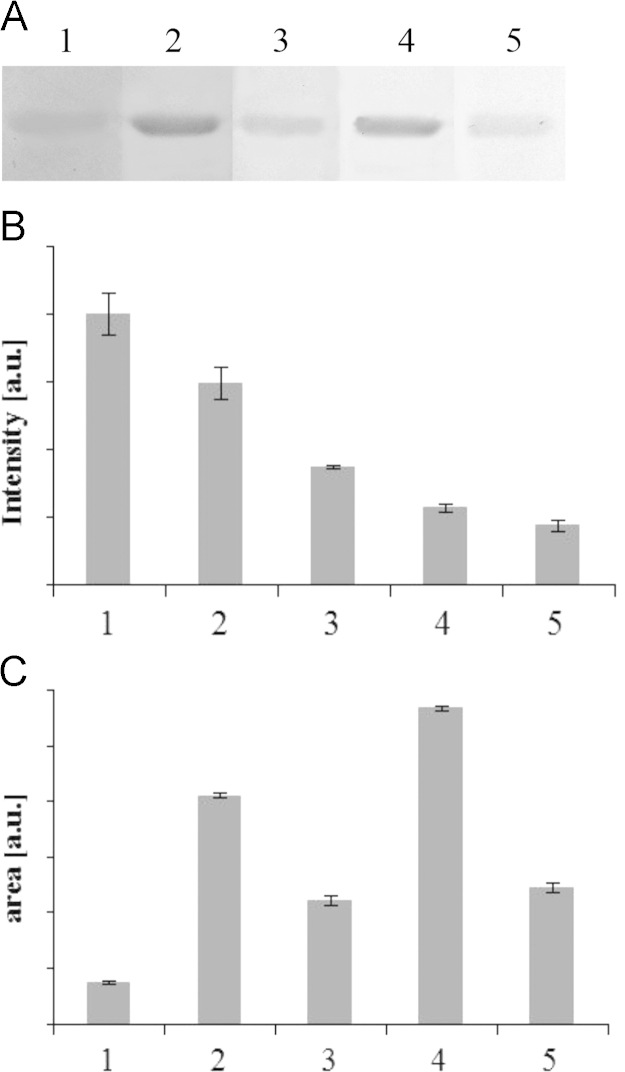
Effect of mediators on laccase-mediated destaining of CBBR-stained polyacrylamide gels. Destaining of polyacrylamide gels was conducted at 25 °C with 20 U mL^−^^1^ laccase and a mediator for 2 h. Lanes 1–5 correspond to ACE (20 μM), SYA (20 μM), HBT (20 μM), ABTS (2 μM) and SYD (20 μM).

**Fig. 2:**
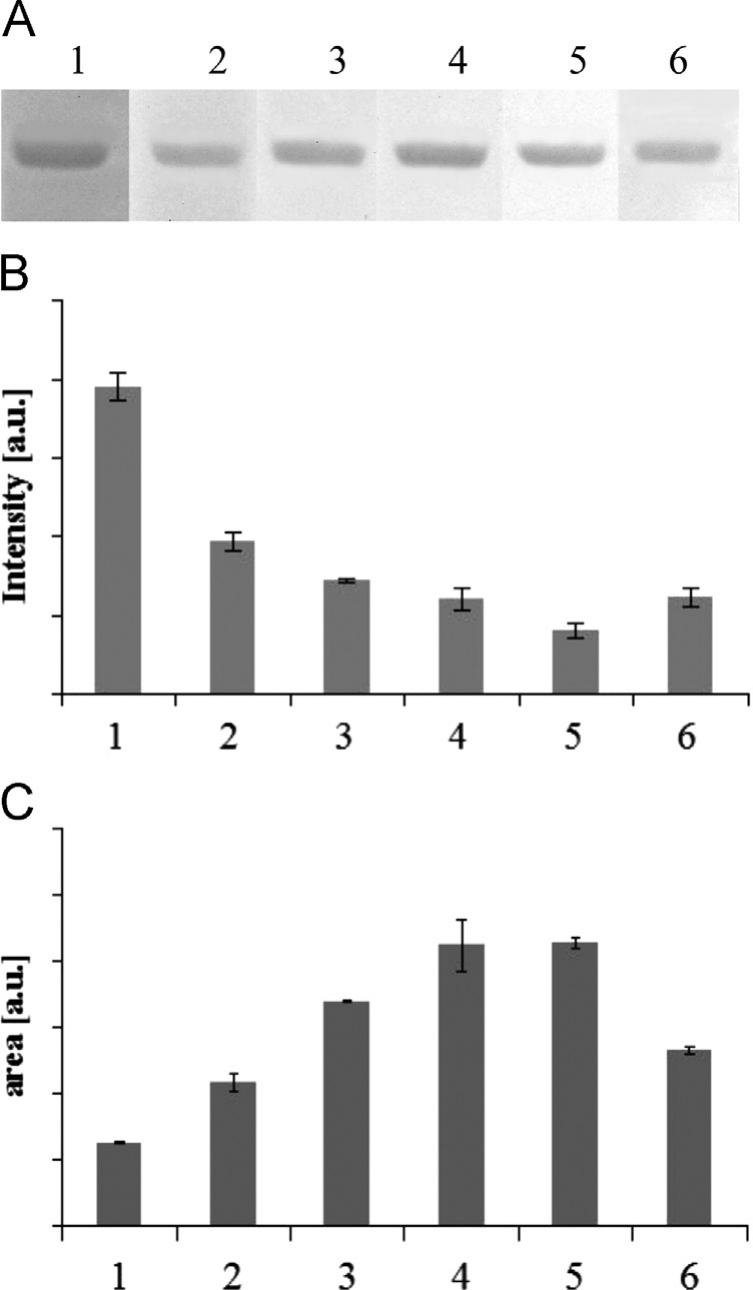
Effect of temperatures on laccase/ABTS-mediated destaining of polyacrylamide gels. Destaining of polyacrylamide gels was conducted at 10–45 °C with 20 U mL^−^^1^ laccase and 2 μM ABTS for 2 h. Lanes 1–6 correspond to 10, 18, 25, 30, 37, 45 °C.

**Fig. 3:**
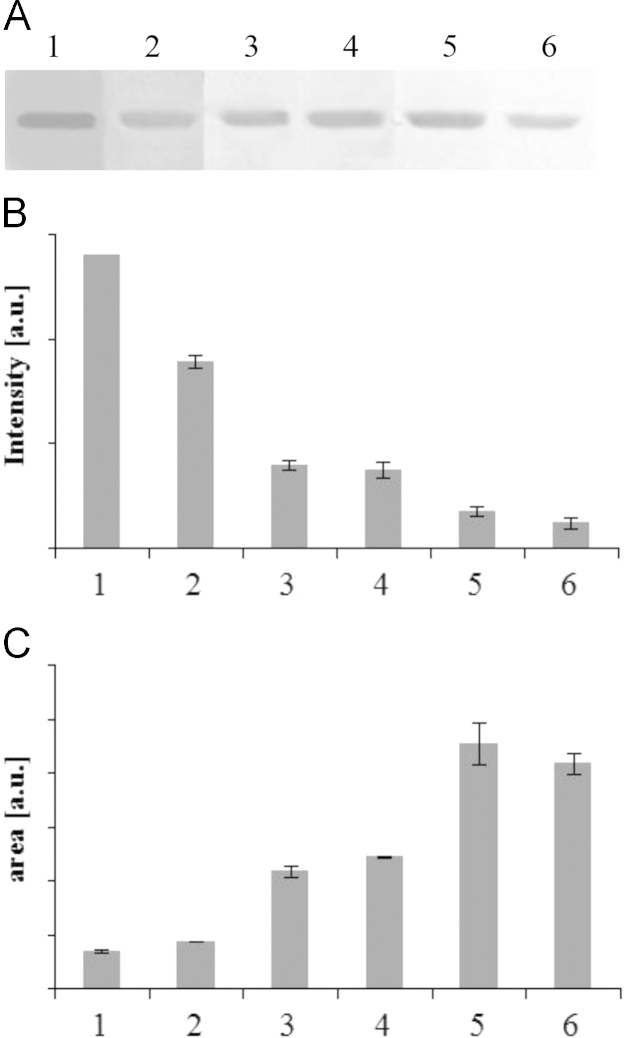
Effect of enzyme activity on destaining of polyacrylamide gels. Destaining of polyacrylamide gels was conducted at 37 °C with different enzyme activities (3–18 U mL^−1^) and 2 μM ABTS for 2 h. Lanes 1–6 correspond to 3, 6, 9, 12, 15, 18 U mL^−^^1^.

**Fig. 4:**
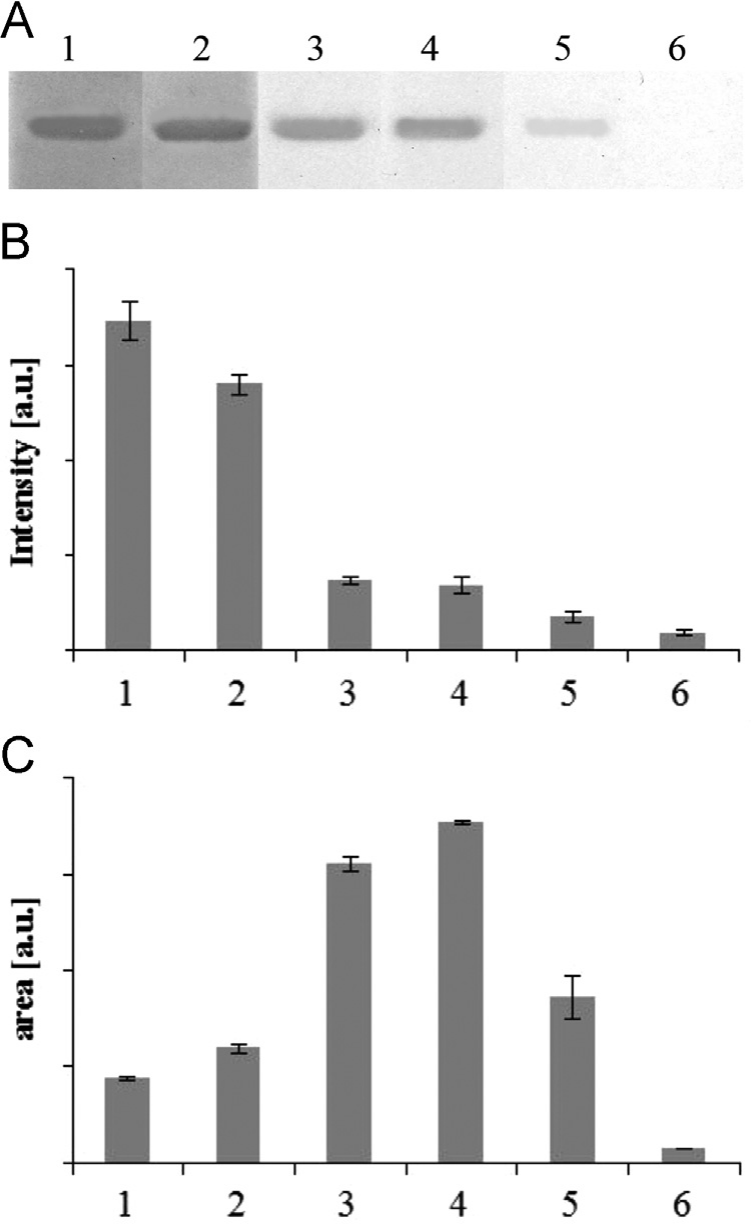
Effect of ABTS concentration on destaining of polyacrylamide gels. Destaining of polyacrylamide gels was conducted at 37 °C with 15 U mL^−1^ laccase and ABTS (0.5–4 μM) for 2 h. Lanes 1–6 correspond to 0.5, 1, 1.5, 2, 3, 4 μM.

**Fig. 5:**
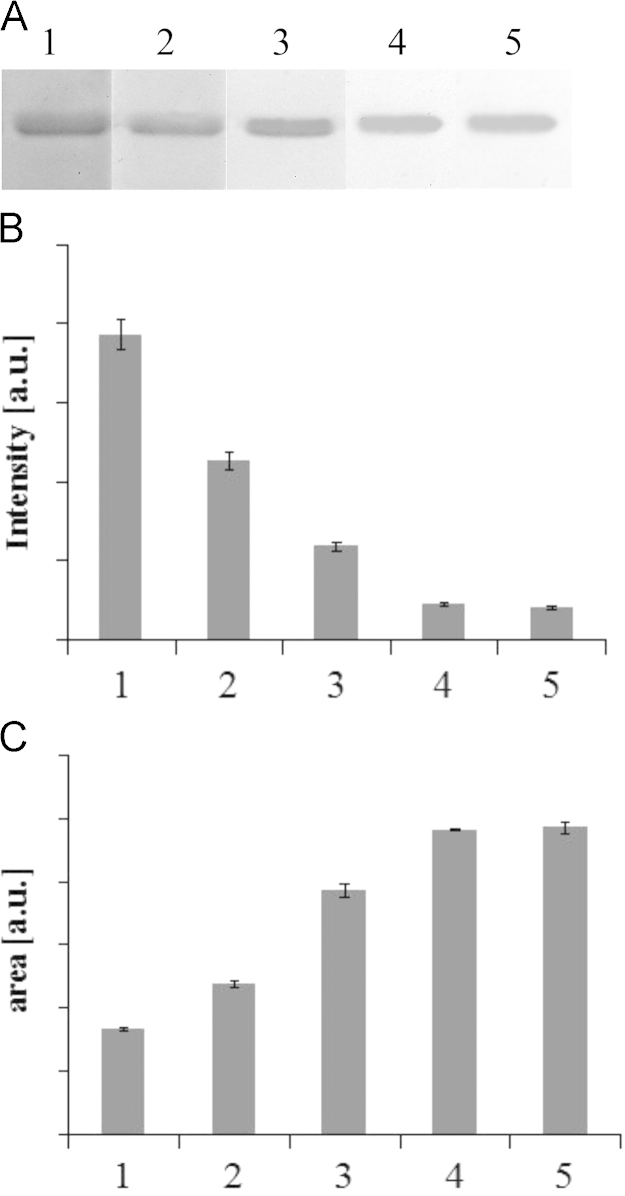
Effect of incubation time on destaining of polyacrylamide gels. Destaining of polyacrylamide gels was conducted at 37 °C with 15 U mL^−^^1^ laccase and 2 μM ABTS for 0.5–3 h. Lanes 1–5 correspond to 0.5, 1, 1.5, 2, 3 h.

Here, we exemplified the application of laccase in destaining of CBBR-stained polyacrylamide gels by using laccase from *Cerrena* sp. HYB07 [2], and various parameters were evaluated based on gel background intensity and protein band signals after destaining. Among the laccase mediators tested, 2,2′-azino-bis (3-ethylbenzothiazoline-6-sulfonate) (ABTS) resulted in low gel background intensity and strong protein signal (Fig. 1). With ABTS as the mediator, effect of temperature, laccase activity, ABTS concentration and destaining time on gel destaining was successively studied (Fig. 2, Fig. 3, Fig. 4, Fig. 5). The optimal temperature, laccase activity, ABTS concentration and destaining time were determined to be 37 °C, 15 U mL^−1^, 2 μM and 2 h, respectively.

## 2 Experimental design, materials and methods

Fermentation of *Cerrena* sp. HYB07 was conducted in a 5 L fermenter for 7 d as previously described [1]. Fermentation broth was harvested by pressure filtration and diluted in distilled water for polyacrylamide gel destaining. Laccase activity was assayed with ABTS (Sigma-Aldrich) as the substrate at pH 3.0 and 40 °C. One unit of enzyme activity (*U*) was defined as the amount of laccase required to oxidize 1 μmol ABTS in 1 min.

Bovine serum albumin (BSA) at 500 ng was separated by SDS-PAGE by using a Mini-PROTEAN Tetra Cell (BioRad, USA) on 5% stacking gels and 12% separating gels (1 mm thick). Staining solution contained 0.05% (w/v) CBBR (Sangon Biotech, Shanghai, China) in distilled water. Unless otherwise stated, the gel was stained by boiling in the staining solution for 1 min and destained in 50 mL destaining solution with shaking. The destaining solution was not changed during destaining. Gel background intensity and protein band areas were measured with ImageJ [3].

To choose the suitable mediator for laccase-mediated destaining of CBBR-stained polyacrylamide gels, different mediators were added to the destaining solution (distilled water supplemented with 20 U mL^−1^ laccase). Acetosyringone (ACE), syringic acid (SYA), 1-hydroxybenzotriazole (HBT) and syringaldehyde (SYD) were used at the final concentration of 20 μM, and ABTS at 2 μM at 25 °C.

Next, effect of temperatures on laccase/ABTS-mediated destaining of polyacrylamide gels was studied. With 20 U mL^−1^ laccase and 2 μM ABTS, destaining was carried out at 10–45 °C for 2 h. Effect of enzyme activity on destaining of polyacrylamide gels was analyzed by using 3–18 U mL^−1^ laccase with 2 μM ABTS at 37 °C for 2 h. Best ABTS concentration was selected by observing the effect of 0.5–4 μM ABTS on destaining with 15 U mL^−1^ laccase at 37 °C for 2 h. Effect of incubation time on destaining of polyacrylamide gels was determined by carrying out gel destaining at 37 °C with 15 U mL^−1^ laccase and 2 μM ABTS for 0.5–3 h.
