# Supplementary material for: Optimal parameters for laccase-mediated destaining of Coomassie Brilliant Blue R-250-stained polyacrylamide gels
Source: Data Brief. 2016 Jan 29;7:1–7. doi: 10.1016/j.dib.2016.01.029 (PMC4761628; doi:10.1016/j.dib.2016.01.029)
Supplement: Supplementary file 1 — Supplementary material [file mmc1.docx]

**Conflicts of interest**

None.
